# Mathematical Modeling of Radiofrequency Ablation for Varicose Veins

**DOI:** 10.1155/2014/485353

**Published:** 2014-12-18

**Authors:** Sun Young Choi, Byung Kook Kwak, Taewon Seo

**Affiliations:** ^1^Department of Radiology and Medical Research Institute, School of Medicine, Ewha Womans University, 1071 Anyangcheon-ro, Yangcheon-gu, Seoul 158-710, Republic of Korea; ^2^Department of Radiology, College of Medicine, Chung-Ang University, 102 Heukseok-ro, Dongjak-gu, Seoul 156-755, Republic of Korea; ^3^Department of Mechanical and Automotive Engineering, Andong National University, 388 Songchun-dong, Andong 760-749, Republic of Korea

## Abstract

We present a three-dimensional mathematical model for the study of radiofrequency ablation (RFA) with blood flow for varicose vein. The model designed to analyze temperature distribution heated by radiofrequency energy and cooled by blood flow includes a cylindrically symmetric blood vessel with a homogeneous vein wall. The simulated blood velocity conditions are *U* = 0, 1, 2.5, 5, 10, 20, and 40 mm/s. The lower the blood velocity, the higher the temperature in the vein wall and the greater the tissue damage. The region that is influenced by temperature in the case of the stagnant flow occupies approximately 28.5% of the whole geometry, while the region that is influenced by temperature in the case of continuously moving electrode against the flow direction is about 50%. The generated RF energy induces a temperature rise of the blood in the lumen and leads to an occlusion of the blood vessel. The result of the study demonstrated that higher blood velocity led to smaller thermal region and lower ablation efficiency. Since the peak temperature along the venous wall depends on the blood velocity and pullback velocity, the temperature distribution in the model influences ablation efficiency. The vein wall absorbs more energy in the low pullback velocity than in the high one.

## 1. Introduction

Contraction of limb muscles forces up venous blood to the heart while walking. Venous leaflet valves prevent the blood from refluxing against gravity. If the elasticity of vein decreases and the leaflet valves work improperly, the blood will then flow backward and the veins will enlarge. This disease is called venous insufficiency. “Varicose veins” are used when the venous insufficiency occurs in the superficial veins of the legs, that is, the great saphenous veins (GSV) and small saphenous veins (SSV) in the lower limbs [1–3]. The varicose veins are fairly easy to identify because they protrude or bulge from under the skin.

The conventional surgical treatments for varicose veins include surgical ligation and stripping of the great saphenous veins. Endovenous laser ablation (EVLA) and radiofrequency ablation (RFA) have been developed as alternatives to surgery because they are minimally invasive. Both endovenous laser ablation and radiofrequency ablation use thermal energy in the form of either laser or radiofrequency energy for closure of blood vessel. Endovenous laser ablation and radiofrequency ablation are designed to ablate varicose veins by introducing heat-induced catheter. Both methods are better than the traditional vein stripping because their success rate is much higher. The clinical results [4–6] have reported that radiofrequency ablation and endovascular laser ablation had no difference in occlusion rate. However, the thermal damage to normal tissue from radiofrequency was less likely than that from the laser light for endovascular laser ablation. Radiofrequency ablation had advantage in that radiofrequency ablation produced less bruising after operation than that of endovascular laser ablation [4].

Besides endovenous ablation of varicose vein, radiofrequency ablation has been used in thermal ablation of liver or kidney tumors, as well as ventricular tachycardia. In various applications of radiofrequency ablation, simulation of thermal effects has been performed by mathematical modeling. Computer models for radiofrequency ablation of endocardium for ventricular tachycardia have been developed with thermal damage function to analyze the extent of the lesion [9–12]. In hepatic tumor ablation, Barauskas et al. [13] presented the character of ablation processes with high frequency electrical current and developed a mathematical model of radiofrequency ablation in liver tissues. Panescu et al. [11] studied the effects of tissue-electrode angle on temperature distribution and of blood flow on current density distribution during radiofrequency ablation. They found that the insulating coating layer over the junction with catheter body decreases the chance of charring and coagulation. Tungjitkusolmun et al. [12] conducted computer simulation to calculate the temperature distribution during radiofrequency ablation in cardiac tissue using ANSYS [14].

Radiofrequency ablation uses the heat generated from high frequency alternating current in the range from 350 to 500 kHz [13]. In endovenous radiofrequency ablation, radiofrequency catheter is inserted into the vein under ultrasound guidance. At an electrode of the catheter, the heat ablates surrounding abnormal vein. If vessel wall temperature is raised above 323 K, cell damage cannot be recovered and coagulation around electrode can occur [7, 15].

Mathematical model for varicose vein may be a useful tool to evaluate the effects of blood flow rates, thermal energy generated from radiofrequency wave, and the occlusion of vein [12, 16]. However, the mathematical modeling of radiofrequency ablation in varicose vein has not been proposed to date. Thus, our aim in this study will be to examine the impact of radiofrequency heat generated in the electrode on blood and vessel wall. Computer simulations for radiofrequency ablation will be conducted to quantify the effect of the heat generated from the electrode and to calculate the temperature profiles in lumen and vessel wall.

## 2. Formulation of the Problem

### 2.1. Geometric Model

**Figure 1:**
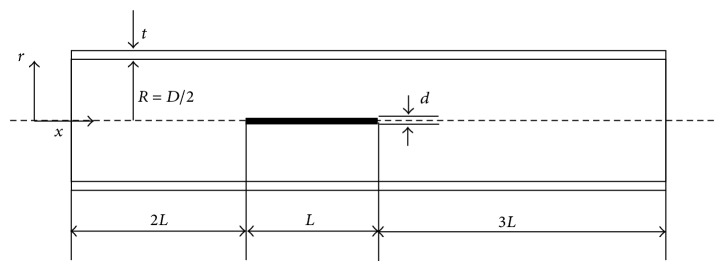
Schematic diagram of model used for simulation: *D* is the vessel diameter (2*R*), *d* is the diameter of electrode, *L* is the length of the electrode, and *t* is the vessel wall thickness.

For the simplification of the geometric model, we ignored the valve leaflets in vein. As shown in Figure 1 the geometry of the vein and electrode has been simplified for half of the vein and tissue. In the study the geometric domains are composed of the lumen and the vessel wall. The lumen is 3 mm in diameter with 60 mm length, while the vein wall is 0.4 mm in thickness with the tunica intima at the interface between blood and vessel wall and tunica adventitia. The electrode is 0.4 mm in diameter with 10 mm length, which is a unitary transformation for 0.4064 mm. The electrode is positioned in the middle of lumen and 20 mm apart from inlet region as shown in Figure 1. In the geometry we assumed that the RF power was supplied to the electrode to conduct the radiofrequency ablation until there is an increase in uncontrolled impedance. In the simulation we assumed that the electrode moves continuously with a constant pullback velocity against the blood flow direction.

### 2.2. Governing Equations and Simulations

In fluid flow, the Reynolds number (Re) is the nondimensional quantity that is defined as the ratio of inertial force to viscous force. If Reynolds number is lower than 2,300, the laminar flow occurs. In this case viscous forces are dominant in a flow regime. For high Reynolds number higher than 2,300, the turbulent flow occurs and is dominated by inertia force. The maximum and minimum Reynolds number in the study were 31.05 and 10.35, respectively, and the average Reynolds number was 20.7. Therefore we assumed the blood flow to be laminar.

We solved the following equations with the moving electrode to understand blood flow and temperature distributions in the varicose vein domain.

 Continuity equation is
  (1) $$\begin{array}{c} \nabla \cdot \vec{u}=0. \end{array}$$
 Momentum equation is
  (2) $$\begin{array}{c} \frac{\partial \vec{u}}{\partial t}+\vec{u}\cdot \nabla \vec{u}=-\frac{1}{\rho_{b}}\nabla p+\frac{\mu }{\rho_{b}}\nabla^{2}\vec{u}, \end{array}$$

where $\vec{u}$ (m/s), *ρ*_*b*_  (kg/m^3^), and *μ* (kg/m*·*s) denote blood velocity vector, blood density, and blood dynamic viscosity, respectively. The first term in (2) represents time dependent acceleration, and the second term is time independent convective acceleration of the flow field with respect to space.

We assumed the vein wall to be homogeneous and isotropic, while the blood flow was unaffected by the heat transfer. Further, the temperatures were influenced by blood flow characteristics. We also assumed the heat transfer between the electrode and the tissue as well as the metabolic internal energy in the study to be neglected [15]. There was a problem as heat diffusion in the vessel wall was subjected to radiofrequency electrical energy, while heat loss occurred by blood flow and thermal convection. The heat process for radiofrequency ablation was numerically solved using bioheat transfer equation given by (1) [12, 17, 18]:

(3) $$\begin{array}{c} \frac{\partial T}{\partial t}+\vec{u}\cdot \nabla T=\frac{K_{w}}{\rho_{w}C_{pw}}\nabla^{2}T+\frac{Q_{\text{RF}}}{\rho_{w}C_{pw}}, \end{array}$$

where $\vec{u}$ is the blood velocity in the lumen and *T* is the temperature of vein wall. *ρ*_*w*_ (kg/m^3^) equals the density of vein wall, *C*_*pw*_ (J/kg*·*K) is the constant specific heat, *K*_*w*_ (W/m*·*K) is the thermal conductivity, and *Q*_RF_ (W/m^3^) is RF energy generated in the tissue. Convection occurs in the lumen in (3) only when blood flows. The second term in (3) refers to transfer of heat with blood flow. In the study the process of transfer of heat from blood flow to vein wall is not only transfer of heat by blood flow, but also diffusion of heat shown by the third term.

Table 1 [8] lists the material properties used in this study. Even if the thermal properties of vessel wall such as specific heat and thermal conductivity depend on temperature [12], we assumed them to be constant in the simulation.

In general, blood is composed of red blood cell (RBC), white blood cell (WBC, leukocytes), and platelets suspended in a fluid medium. Even though blood viscosity is considered a non-Newtonian fluid with shearing thinning properties [19], blood has a Newtonian behavior with viscosity of 1.6 × 10^−3^ N*·*s/m^2^ [20]. This study models blood as a generalized Newtonian fluid using Carreau-Yasuda model [21, 22]:

(4) $$\begin{array}{c} \frac{\mu -\mu_{\infty }}{\mu_{0}-\mu_{\infty }}={\left[1+{\left(\lambda \dot{\gamma }\right)}^{a}\right]}^{\left(n-1\right)/a}, \end{array}$$

where *μ*_*∞*_ is the viscosity at infinite shear rate, 0.0035 kg/m*·*s, *μ*_0_ is the viscosity at zero shear rate, 0.16 kg/m*·*s, nondimensional power index *n* = 0.2128, *a* = 0.64, and the relaxation time *λ* = 8.2 s. $\dot{\gamma }$ (rad/s) represents the shear rate at which a shearing deformation is applied to blood.

We assumed the blood flow as a steady laminar flow and the velocity profile at the inlet to be uniform. The study investigated the effects of blood flow and vessel wall by radiofrequency heat generated in the electrode under the conditions of blood velocities of 0, 1, 2.5, 5, 10, 20, and 40 mm/s. The temperature variations in the model within blood and vessel wall due to the thermal energy generated from the electrode in the middle of lumen can be modeled by the concept of conjugate heat transfer (CHT). The conjugate heat transfer corresponds with the combination of heat transfer in blood vessel and heat transfer in blood. In blood vessel, the heat conduction dominates, while the heat convection is dominant in blood. We solved the governing equations using the commercial computational fluid dynamic code CFX (ANSYS 14.5, Canonsburg, PA). CFX is a finite-volume code in which the flow equations are discretized for each cell in the system. A high resolution advection scheme is applied to solve the discretized equations.

**Figure 2:**
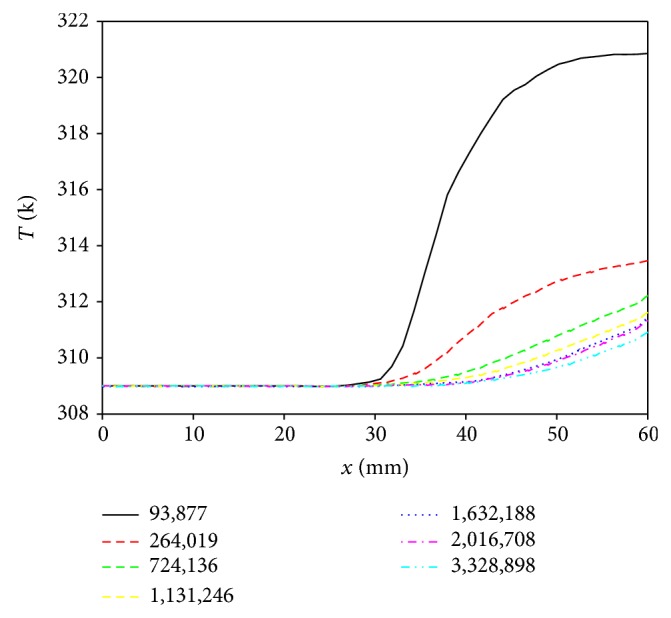
Temperature distributions along the interface between blood and vessel wall to evaluate the mesh quality.

The study performed all simulations using Intel Xeon CPU E5-1650 at 3.4 GHz (32 GB of RAM) personal computer running 64-bit Windows 7. All solutions presented have been verified to be mesh independent; increasing the mesh density yields velocities that are within 1% of those shown in Figure 2. All simulation results in the study had the size of the root mean square (RMS) residual of less than 1 × 10^−05^ with adopted numbers of meshes, about 1.6 million cells.

### 2.3. Boundary Conditions

We considered the vessel walls to be rigid, so our model excluded the elastic movement of vein. The thermal boundary condition at the tunica adventitia had zero heat flux. We assumed the surfaces of the electrode to be a source of heat to keep the constant temperature. The thermal energy transferred from heat source could reach the interfacial plane and the energy could be conducted through the vessel wall. We assumed the temperature of incoming blood to have human body temperature (36°C) and applied the zero heat flux boundary condition at the exit of the blood flow. We assumed a uniform flow with uniform blood velocity in the axial direction at the inlet, while assuming the wall boundaries to satisfy no-slip condition. We assumed the velocity boundary conditions at the inlet for *U* = 0, 1, 2.5, 5, 10, 20, and 40 mm/s. We calculated the preset temperature at the electrode-blood interface due to RF wave energy at 358 K (85°C) [23]. Outlet boundary is sufficiently far from the electrode tip (30 mm) in order to prevent interface with the lesion process and to keep to the body temperature of 36°C.

## 3. Results and Discussion

**Figure 3:**
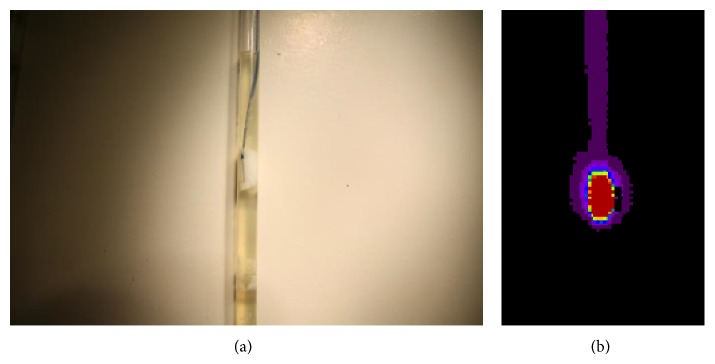
(a) Egg white coagulation* in vitro* experiment and (b) thermal imaging for 5 mm diameter transparent plastic straw filled with egg white during egg white coagulation.

Figure 3(a) shows* in vitro* ablation of egg white in tube. The electrode catheter/guide wire system functionality has been confirmed by the ability to coagulate egg white. The study conducted the coagulation* in vitro* study with electrode catheter with 10 mm circular cathode and 5 mm electrode of guide wire at room temperature [24]. We observed that the coagulations from the electrode were gradually spreading. The study performed the thermal imaging for egg with coagulation in Figure 3(b) at the transparent plastic straw filled with egg white using a digital IR thermal imager (IRIS-XP, Medicore, Seoul, Korea).

**Figure 4:**
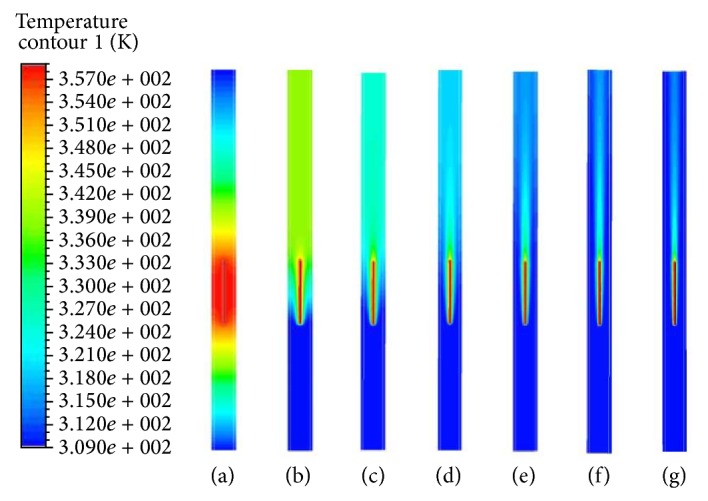
Temperature distributions in symmetry plane for various blood velocities for electrode temperature 358 K. (a) *U* = 0. (b) *U* = 1 mm/s. (c) *U* = 2.5 mm/s. (d) *U* = 5 mm/s. (e) *U* = 10 mm/s. (f) *U* = 20 mm/s. (g) *U* = 40 mm/s.

Figure 4 presents temperature profiles for blood inlet velocity *U* = 0, 1, 2.5, 5, 10, 20, and 40 mm/s. We assumed the electrode as a heat source that emits heat uniformly. This spread heat into the bloodstream homogeneously and the vessel wall through the thermal conduction and convection. With the blood velocity increasing, Figure 4 notes that less heat caused by the energy emitted from the electrode was transmitted to the vessel wall.

If the tissue temperature becomes above about 313 K (40°C), then thermal damage can occur to tissues [13, 15, 25]. As blood velocity increases, perfusion of heat loss also increases due to the blood convection cooling effect. As a result, tissue temperature decreases. If blood flow is stagnant, the temperature can be uniformly distributed in the whole domain (see Figure 4(a)). As seen in Figure 4, the temperature patterns are strongly dependent on the blood velocity. Since the amount of energy transfer for the ablation varies depending on the lesion size, it is important to control the blood flow rate to prevent the tissue damage. From the results, the lower the blood velocity, the higher the temperatures in the vessel wall and the more the damage to the tissue. As seen in Figure 4, the high temperature occurred at the tip of electrode. The generated radiofrequency energy induced a temperature rise of the blood in the lumen and led to an occlusion of the blood vessel.

**Figure 5:**
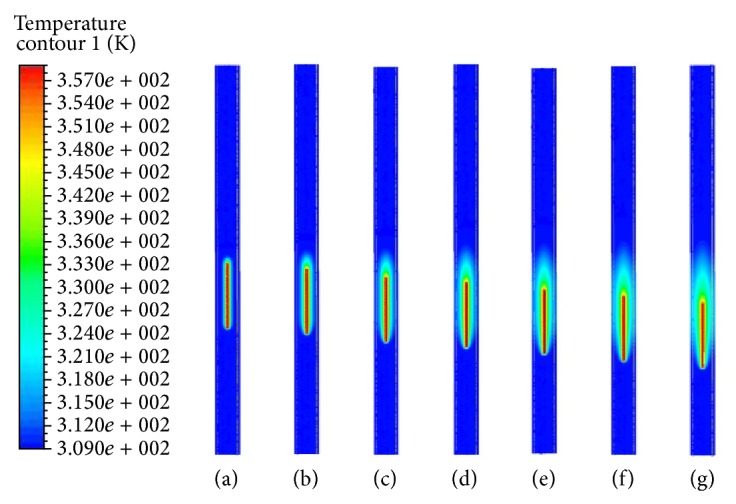
Temperature distributions in symmetry plane at seven distinct times during pulling back of electrode at constant velocity of 1 mm/s (in stagnant flow situation). (a) *t* = 0.5 s. (b) *t* = 1.5 s. (c) *t* = 2.5 s. (d) *t* = 3.5 s. (e) *t* = 4.5 s. (f) *t* = 5.5 s. (g) *t* = 6.5 s.

**Figure 6:**
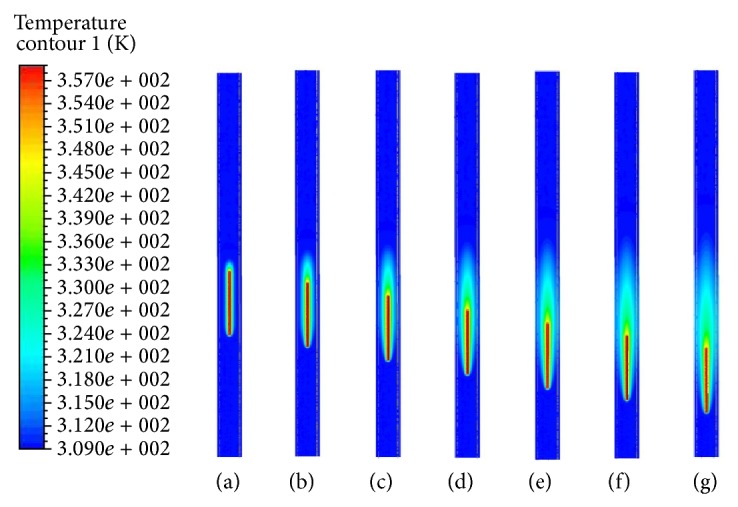
Temperature distributions in symmetry plane at seven distinct times during pulling back of electrode at constant velocity of 2 mm/s (in stagnant flow situation). (a) *t* = 0.5 s. (b) *t* = 1.5 s. (c) *t* = 2.5 s. (d) *t* = 3.5 s. (e) *t* = 4.5 s. (f) *t* = 5.5 s. (g) *t* = 6.5 s.

If the electrode is continuously pulled back with a certain velocity for 7 seconds, Figures 5 and 6 show the results of temperature distributions at the seven distinct times for stagnant blood. As time increases, the generated heat induces a rise in temperature of the lumen and vein wall around electrode and eventually leads to the obliteration of the vein. Comparing the results of Figures 5 and 6, we can see that the temperature distribution in the domain has a similar fashion. However, the vein wall absorbs more energy in the low pullback velocity than in the high one in the comparison of Figures 5 and 6 [17]. Consequently, the blood velocity, in addition to the pullback velocity, can also affect the convection effect. Additionally when radiofrequency ablation begins to heat, the pullback velocity limits the heat transfer region.

As time passes, the region under the effect of temperature variation expands up to 2.9 times the initial situation (refer to Figures 6(a) and 6(g)). Radiofrequency energy expands to the vessel wall by the diffusion with time. Since blood will flow with the relative velocity against the electrode pullback velocity, the thermal boundary layer develops in the region of the electrode and gradually diffuses toward the vessel wall. Since radiofrequency energy cannot be delivered to the blood vessel zone far from the electrode, the electrode should be positioned at the end of the site to be closed during radiofrequency ablation therapy for varicose vein. The radiofrequency energy generated from the electrode diffuses homogeneously to the lumen and vessel wall and temperature rises to the electrode surface temperature with time as shown in Figures 5 and 6. We assumed a constant temperature at the electrode surface, so the blood did not boil during radiofrequency ablation procedure.

**Figure 7:**
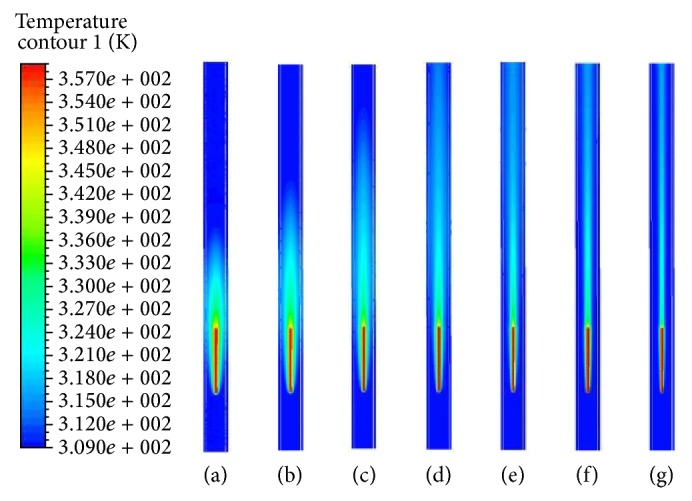
Temperature distributions in symmetry plane at *t* = 5.5 s during pulling back of electrode at constant velocity of 2 mm/s (in various blood flow velocities situation). (a) *U* = 0. (b) *U* = 1 mm/s. (c) *U* = 2.5 mm/s. (d) *U* = 5 mm/s. (e) *U* = 10 mm/s. (f) *U* = 20 mm/s. (g) *U* = 40 mm/s.

Figure 7 illustrates the temperature distributions in the symmetry plane for various blood velocities at time *t* = 5.5 s. The more the increase in blood velocity, the smaller the transferred region of RF energy. The maximum temperature of approximately 330 K or above occurs at the tip of the electrode. When blood velocity is greater than 2.5 mm/s, RF energy cannot be delivered to the vein wall. This is because energy transfer by convective flow is stronger than the one by diffusion. Since the convective blood flow is deprived of the thermal energy of the electrode, it is hard to apply RF ablation of varicose veins if blood velocity is high.

**Figure 8:**
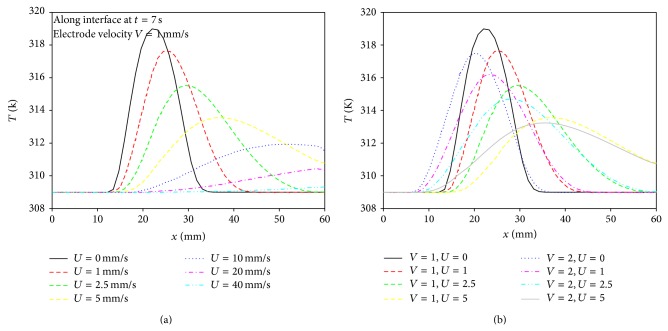
Temperature distributions along the interfacial surface in the axial direction between blood and vessel wall for various blood velocities at *t* = 7 s.

Temperature profiles along the interfacial surface between lumen and vessel wall for various blood velocities at time *t* = 7 s are shown in Figure 8(a). The peak temperature occurs at the tip of the electrode and decreases as the blood velocity increases. As the blood velocity increases, the location of the peak velocity moves to the outflow region. As the blood velocity increases more and more, the energy by the convective heat transfer becomes larger than energy by conductive heat transfer. The region that is influenced by temperature in the case of the stagnant flow occupies approximately 28.5% of the whole geometry. However, the region that is influenced by temperature in the case of continuously moving electrode against the flow direction is about 50%. As the blood velocity increases from 0 to 1, 2, 2.5, and 5 mm/s, the region under the influence of temperature expands between 13.6% and 37.8% depending on the blood velocity compared with the region for the stagnant flow condition. The peak temperature along the venous wall is between 309 K (36°C) and 316.8 K (43.8°C) depending on the blood velocity. Thus, as the blood velocity increases, the peak temperature decreases by the cooling effect of the blood flow and causes lower ablation efficiency.

Figure 8(b) represents the temperature profiles along the interfacial line for blood velocity *U* = 0, 1, 2.5, and 5 mm/s in the case of pullback velocity *V* = 1 and 2 mm/s. The peak temperature gradually decreases depending on the blood velocity. The peak temperature decreases from 319 K for stagnant flow to 313.8 K for blood velocity *U* = 5 mm/s in the case of pullback velocity *V* = 1 mm/s, while the peak temperature is from 317 K to 313.3 K in the case of pullback velocity *V* = 2 mm/s. As a result, the movement of the electrode induces the flow disturbance in the lumen, and heat moves faster downstream by the convection effect.

**Figure 9:**
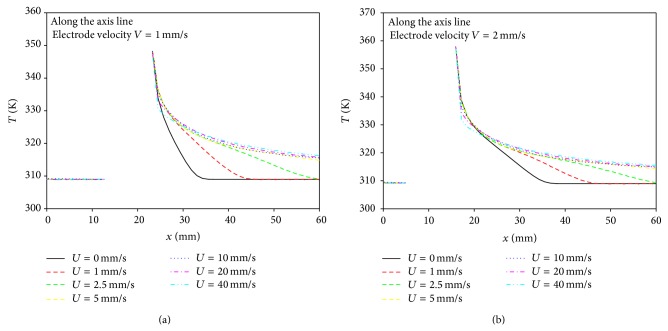
Temperature distributions along the interfacial surface in the axial direction for various blood velocities at electrode velocities (a) *V* = 1 mm/s and (b) *V* = 2 mm/s.

Figure 9 represents temperature distributions along the axis for various blood velocities at time *t* = 7 s in the cases of both pullback velocities of electrode of *V* = 1 mm/s and 2 mm/s. In Figure 9, the temperature decreases gradually and reaches a constant value in downstream in the case of high blood velocity, while temperature decreases abruptly in the case for low blood velocity. As a result, since temperature increase in the lumen could be prevented when the pullback velocity of electrode increases, it is preferred that the electrode catheter moves to keep a certain blood temperature in downstream region during RFA procedure.

## 4. Conclusion

Our goal in developing this model was to examine the impact in blood and vessel wall by radiofrequency heat generated in the electrode. We conducted computer simulations for radiofrequency ablation to quantify the effect of the heat generated from the electrode. Furthermore, we used a simplified three-dimensional geometry of vein to calculate the temperature profiles in lumen and vessel wall and inputted previously published data regarding the thermal properties of human blood and vein into ANSYS CFX 15 [14].

The study posed some limitations. First, our model was limited to a single-phase laminar flow regime and neglected the venous leaflet valve. Second, we assumed an isotropic tissue in this study. In the future, we will simulate a model that considers the particles in the blood such as red blood cell (RBC) and platelet as well as the venous valve to obtain more physiological results in the impact of radiofrequency energy in the blood and vessel wall.

If venous blood flow is increased, then heat transfer to vein wall is hindered by the convective flow. Thus heat energy cannot reach vein wall, and the efficacy of radiofrequency ablation reduces. During radiofrequency ablation, the best condition to have good efficacy is to apply the stagnant blood flow condition. The result of the study demonstrated that the higher blood velocity led to smaller thermal region and lower ablation efficiency. We observed higher blood and vein wall temperatures on the outflow region than on the inflow region. The result of the study also showed that the temperature increase in the lumen could be controlled when the pullback velocity of electrode increases.

## Figures and Tables

**Table 1:** Material property values used in the study obtained from Mordon et al. (2006) [7] and Agalar et al. (2012) [8].

Property	Symbol	Value
Density of blood	*ρ*_*b*_	1050 kg/m^3^
Dynamic viscosity of blood	*μ*_*b*_	0.0035 kg/m*·*s
Density of vein wall	*ρ*_*w*_	1120 kg/m^3^
Specific heat of blood	*C*_*pb*_	3820 J/kg°C
Specific heat of vein wall	*C*_*pw*_	3780 J/kg°C
Thermal conductivity of blood	*K*_*b*_	0.492 W/m*·*K
Thermal conductivity of vein wall	*K*_*w*_	0.56 W/m*·*K
